# PhyreRisk: A Dynamic Web Application to Bridge Genomics, Proteomics and 3D Structural Data to Guide Interpretation of Human Genetic Variants

**DOI:** 10.1016/j.jmb.2019.04.043

**Published:** 2019-06-14

**Authors:** Tochukwu C. Ofoegbu, Alessia David, Lawrence A. Kelley, Stefans Mezulis, Suhail A. Islam, Sophia F. Mersmann, Léonie Strömich, Ilya A. Vakser, Richard S. Houlston, Michael J.E. Sternberg

**Affiliations:** 1 Centre for Integrative Systems Biology and Bioinformatics, Department of Life Sciences, Imperial College London, London, SW7 2AZ, UK; 2 Computational Biology Program and Department of Molecular Biosciences, The University of Kansas, Lawrence, KS 66045, USA; 3 Division of Genetics and Epidemiology, The Institute of Cancer Research, London, SM2 5NG, UK

**Keywords:** web resource, sequence-structure mapping, human proteome, genetic variants, PDB, Protein Data Bank, VEP, Variant Effect Predictor

## Abstract

PhyreRisk is an open-access, publicly accessible web application for interactively bridging genomic, proteomic and structural data facilitating the mapping of human variants onto protein structures. A major advance over other tools for sequence-structure variant mapping is that PhyreRisk provides information on 20,214 human canonical proteins and an additional 22,271 alternative protein sequences (isoforms). Specifically, PhyreRisk provides structural coverage (partial or complete) for 70% (14,035 of 20,214 canonical proteins) of the human proteome, by storing 18,874 experimental structures and 84,818 pre-built models of canonical proteins and their isoforms generated using our *in house* Phyre2. PhyreRisk reports 55,732 experimentally, multi-validated protein interactions from IntAct and 24,260 experimental structures of protein complexes.

Another major feature of PhyreRisk is that, rather than presenting a limited set of precomputed variant-structure mapping of known genetic variants, it allows the user to explore novel variants using, as input, genomic coordinates formats (Ensembl, VCF, reference SNP ID and HGVS notations) and Human Build GRCh37 and GRCh38. PhyreRisk also supports mapping variants using amino acid coordinates and searching for genes or proteins of interest.

PhyreRisk is designed to empower researchers to translate genetic data into protein structural information, thereby providing a more comprehensive appreciation of the functional impact of variants. PhyreRisk is freely available at http://phyrerisk.bc.ic.ac.uk

## Highlights

• PhyreRisk is a web application that facilitates mapping of human variants onto protein structures.
• PDB and predicted structures are provided for both canonical and isoform proteins.
• Genetic variants can be mapped from genomic and proteomic coordinates.
• PhyreRisk provides total or partial coverage for 70% of human proteins.

## Graphical Abstract

**Figure:**
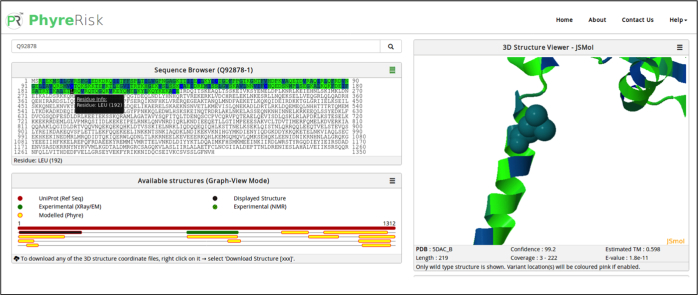

## Introduction

The sheer scale of information from large genome projects such as the UK 100K Genomes Project has led to a formidable challenge of interpreting the functional consequences of genetic variants. Determining the effect of an amino acid substitution on protein structure is central to the interpreting genetic variants [1]. Experimental three-dimensional structures of proteins deposited in Protein Data Bank (PDB) [2], however, cover less than 20% of the human proteome. Homology modeling is a powerful tool for generating a 3D structure in the absence of experimental structure, and several homology modeling servers and databases are available. These include Rosetta [3], I-Tasser [4] and our *in house* program Phyre2 [5]. By means of homology modeling, structural coverage of the human proteome is significantly enhanced reaching 70% [6].

Current tools for mapping of variants using genomic coordinates onto PDB experimental structures are CRAVAT [7] and MuPIT [8]. Similarly, the G2S (Genome to Structure) server provides web APIs for the mapping of UniProt positions onto experimental protein structures [9]. Protein structure resources focusing on genetic variant interpretation in the context of cancer genomics are Cosmic3D [10] and MOKCa [11] that allow mapping of variants reported in the Cosmic database onto experimental 3D structures. Interactome3D [12] and Interactome INSIDER [13] provide structural coverage of protein–protein complexes. Other resources, such as LS-SNP/PDB [14] and SNP2structure [15], have unfortunately not been maintained.

**Fig. 1:**
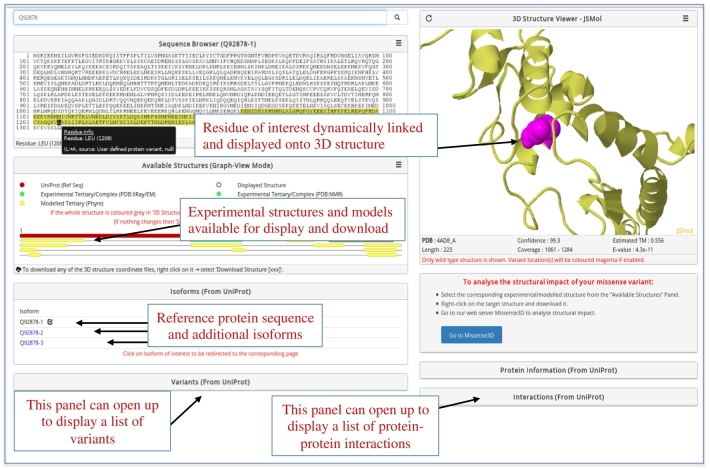
Example of Protein page displayed by PhyreRisk.

The tools that are now available for mapping variants to structures have two major limitations. First, they map to experimental structures but do not support the use of predicted 3D models. Second, they only allow limited use of standard genetic variant input formats, such as Ensembl, VCF, variant identifiers (rs Id) and Human Genome Variation Society notations. The lack of support for input from a wide range of genomic-based coordinates is a major obstacle to use by the genetic-based community. To address these limitations, we developed PhyreRisk (Fig. 1), a user-friendly and publicly accessible “one-stop-shop” web application, specifically designed to bridge genomic, proteomic and structural data, and facilitate mapping of human variants onto protein structures. In addition, the PhyreRisk database providing a comprehensive resource linking human protein sequences to both experimental and Phyre-predicted structures should have applications in a wide range of studies including drug development.

PhyreRisk is available at http://phyrerisk.bc.ic.ac.uk

## PhyreRisk Overview

The combination of the following key features makes PhyreRisk a valuable resource:

• User defined genetic variants can be inputted using both genomic coordinates (human built GRCh37 or GRCh38) or proteomic coordinates.
• The Protein page provides dynamic display of sequence-structure mapping onto experimental and Phyre predicted models (Fig. 1).
• Structural coverage is displayed graphically with coordinates that can be downloaded.
• Sequence and models for canonical and isoforms are provided.

PhyreRisk is implemented in Java. A guided online tutorial is available on the home page to help users navigate and learn how to use PhyreRisk.

### PhyreRisk database and data sources

**Fig. 2:**
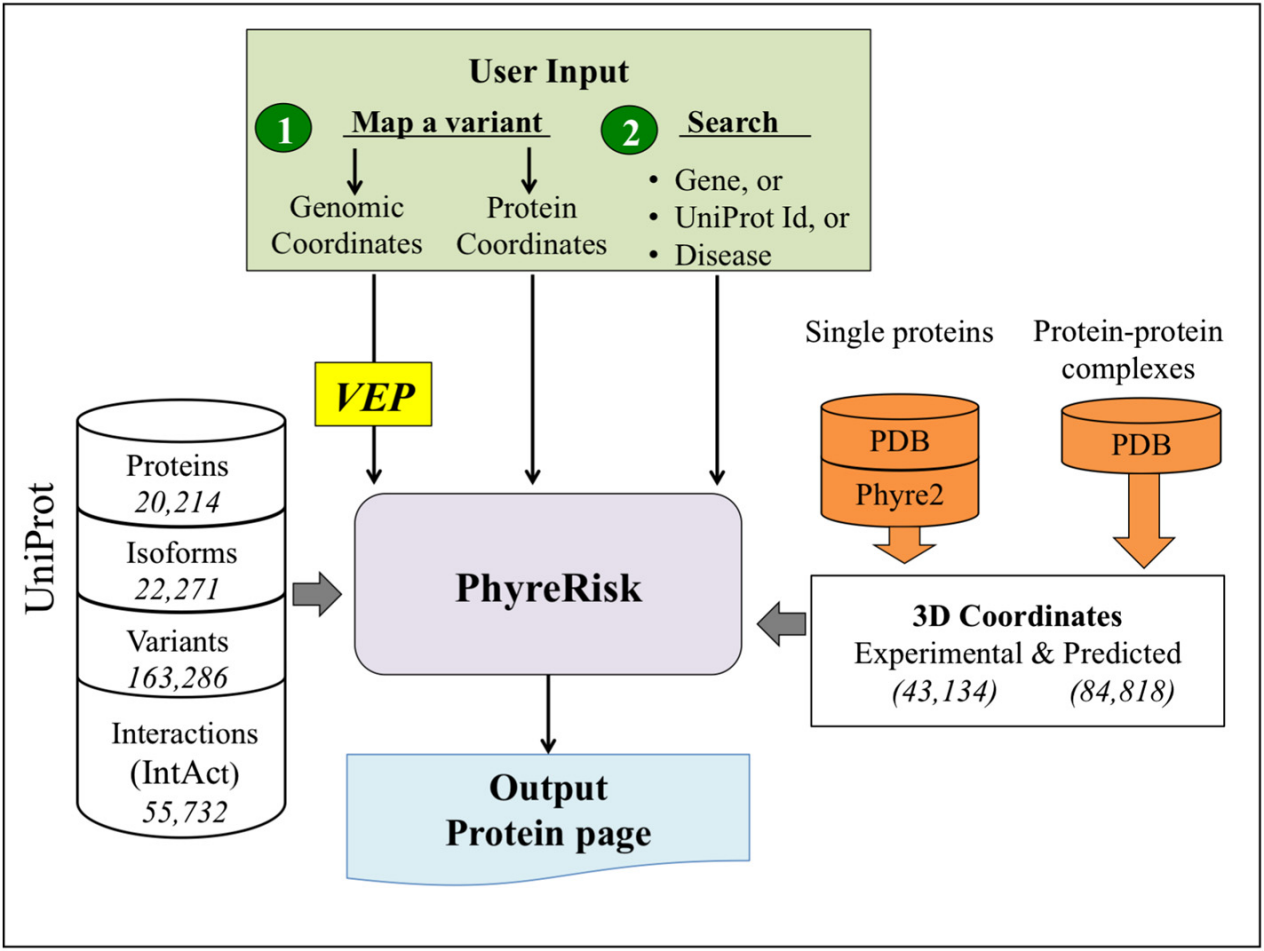
Overview of the PhyreRisk pipeline.

Figure 2 shows the data sources integrated into PhyreRisk. PhyreRisk contains fasta sequences for 20,214 human proteins, which are presented as the canonical forms based on the UniProt [16] database, and for 22,271 protein isoforms (i.e., proteins derived from alternative splicing or the use of alternative promoter or start codons, as per UniProt definition). In addition, 55,732 experimentally derived protein–protein interactions supported by multiple observations from the IntAct [17] database (as per UniProt filtering) are stored in the database. Currently, 163,286 variants from UniProt Humsavar database have been curated. In the current version of PhyreRisk, we have chosen not to store all known human variants catalogued by other databases, such as ExAC [18] and dbSNP [19].

PhyreRisk stores all its information in a postgreSQL relational database. Automatic update of the database is currently manually triggered.

### Structural coverage of canonical sequences and isoforms: experimental structures and models

The greatest strength of PhyreRisk is the structural coverage of the human proteome. The database contains 18,874 experimental structures corresponding to single proteins (tertiary structures) from PDB. Moreover, it stores 84,818 pre-built predicted tertiary structures corresponding to canonical and isoform protein sequences generated using our *in house* Phyre2 software [5]. Overall, PhyreRisk provides structural coverage (partial or complete) for 14,035 (70%) out of 20,214 UniProt canonical protein sequences, of which 7987 proteins are covered by a predicted 3D model.

Because PhyreRisk aims to provide users with as much structural information as possible, especially in terms of the effect of variants on a biological system rather than just a single protein, the database incorporates all 24,260 experimental structures of protein complexes available from PDB. No selection criteria were applied to this dataset. We are working on future development of PhyreRisk to also incorporate predicted 3D coordinates of protein complexes from GWIDD [20].

## The Input Page

The PhyreRisk pipeline can handle three types of input data: (i) the user's own set of variants in genomic coordinates, (ii) the user's own set of variants using amino acid coordinates or (iii) a gene name, UniProt Id or disease name.

### Genomic variants input page

**Fig. 3:**
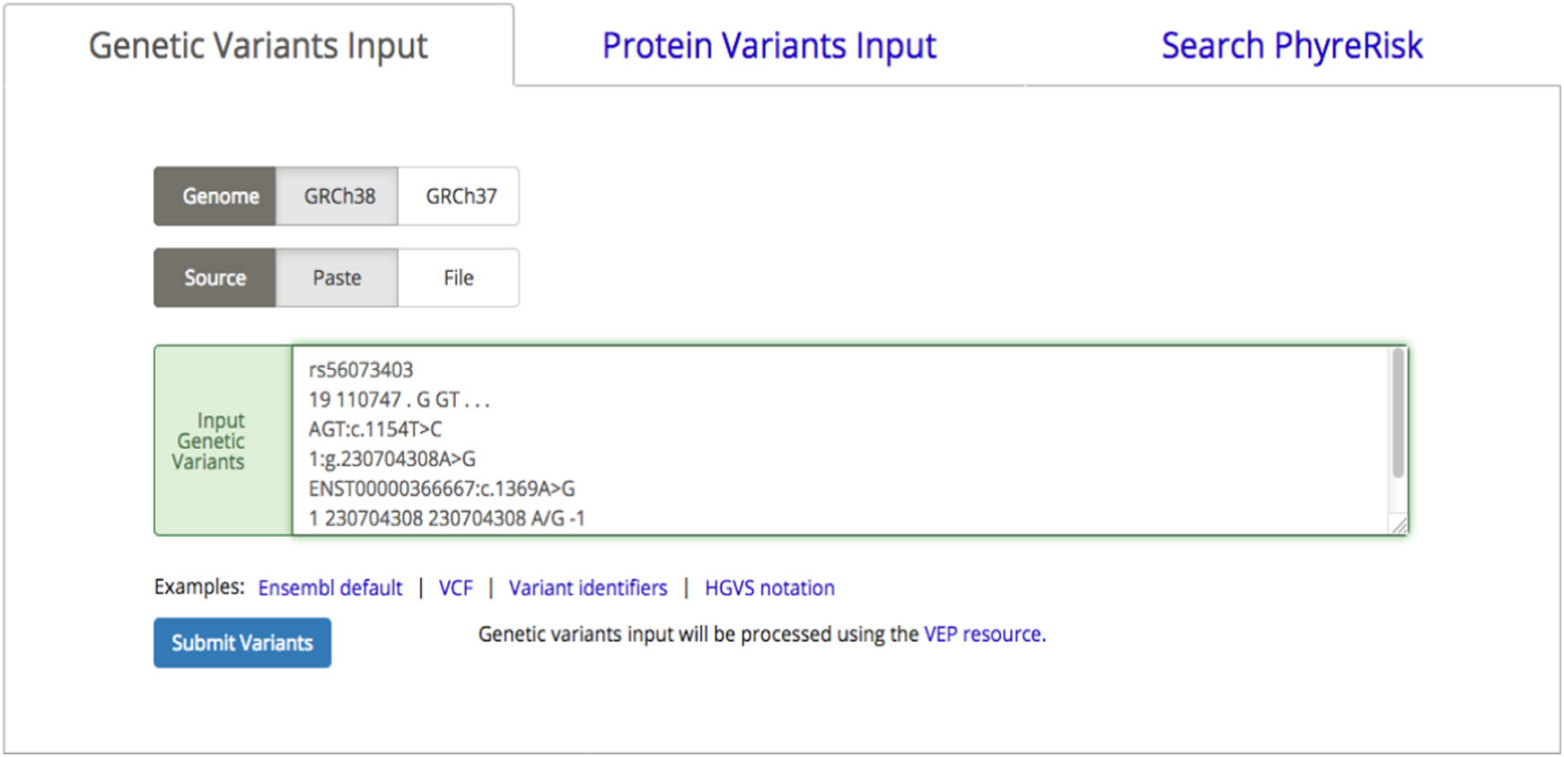
PhyreRisk variant Input page.

Genetic variant coordinates can be described using the most commonly used formats: Ensembl, VCF, variant identifiers (rs Id) or Human Genome Variation Society notations (Fig. 3). One of the strengths of PhyreRisk is that it is a dynamic resource, which supports genome build GRCh37 and GRCh38. In contrast to the static nature of most available 3D mapping tools, such as Cosmic3D [10], which provide a list of genetic variants for which mapping is available, PhyreRisk implements a RESTful Web Service interface to programmatically query the Ensembl Variant Effect Predictor (VEP) [21]. After submitting the variant input, the PhyreRisk Results page appears within seconds and displays, among others, the variant description at protein level, its consequence term (this is the sequence ontology term assigned by VEP to the variant), as well as SIFT [22] and PolyPhen2 [23] *in silico* predictions (Suppl Fig. 1). All the information reported in this page is from VEP at EBI. The Results page provides a link into the PhyreRisk page, corresponding to the protein harbouring the query variants. Within the page, the amino acid under investigation is highlighted on the sequence of the protein and displayed on a 3D structure if experimental or model 3D coordinates are available.

### Protein variant input page

This page can be used to explore missense variants using the amino acid position rather than their genomic coordinates (Suppl Fig. 2). The required information includes the following: the UniProt Id of the protein harbouring the substitution, the position of the wild-type amino acid, the wild-type residue and its substitution. After *submitting*, the Results page is displayed within a few seconds and provides a link to the protein's PhyreRisk page.

### Search page

This page allows a search of any human protein (canonical) in PhyreRisk (Suppl Fig. 3). The search box has the autocomplete functionality enabled. PhyreRisk can be searched using different terms corresponding to the same protein. For example when searching for the low-density lipoprotein receptor adapter protein 1, one can type: (i) the gene name, for example, LDLRAP; (ii) the canonical UniProt ID, for example, Q5SW96; (iii) the UniProt entry name, for example, ARH_HUMAN; or (iv) the extended protein name, for example, low-density lipoprotein receptor adapter protein 1.

The Search page can also be used to search for “diseases.” As an example, searching for “breast cancer” will return a list of all proteins, which according to the UniProt database associated with this disease.

Importantly, when searching for a protein or gene, multiple isoforms (corresponding to different transcripts) may exist. PhyreRisk adopts the UniProt classification to define the “canonical” amino acid sequence (indicated by an asterisk in PhyreRisk), and this is the one displayed by default in the protein page (see “Isoform panel” below for more information on how PhyreRisk handles isoforms).

## The Protein Page

### The Sequence browser

The Sequence browser named Web Alignment Viewer and Editor (WaveJS) was developed *in house* using JavaScript and WebGL. This allows fast, interactive visualization of a protein sequence with its features and annotations. It also enables partial bi-directional communication with the JSmol [24] molecular viewer. A Tooltip is available to display the structural coverage on the fasta sequence or to display variants currently available in the database or supplied by the User. The tooltip also allows choosing how to colour the 3D structure (e.g., from the default gold to cyan).

### Structure selection and 3D Structure viewer panels

The structure selection panel presents in a graphical or list-view mode all the available structures (experimental or model) stored in PhyreRisk. In the graphical-view mode, the protein amino acid sequence is presented as a bar. All available structures are also displayed as horizontal bars underneath the amino acid sequence bar, thus providing an intuitive presentation of the amino acid sequence-structure coverage. Structures are ranked and presented according to their sequence-structure coverage and structure resolution or confidence in template, for experimental structures and models, respectively.

The  3D Structure viewer panel enables the user to graphically visualize the atomic coordinate information for the selected protein structure file. Currently, PhyreRisk supports the 3D molecular viewers, JSmol, 3DMol [25], and NGL [26]. A sequence-structure mapping is performed using the built-in Structure Integration with Function, Taxonomy and Sequence (SIFTS) [27] from ePDB. However, interactive communication with the Sequence browser is so far only implemented for the JSmol viewer. It is anticipated support for bi-directional communication will be extended to NGL and 3DMol in the near future.

At present, the sequence-structure bidirectional communication allows to visualize only one residue at a time. However, the built-in JSmol viewer functions are enabled in the Structure viewer and allow easy visualization and manipulation of residues of interest on the preferred structure (examples and step-by-step guide on how to display two or more residues on the same structure are presented in the Supplementary Material). PhyreRisk is not designed to be used as a molecular viewer. For such a task, we would recommend using molecular viewers, such as Pymol (which has extensive functionality) [28] or EzMol (with guided commands designed for the occasional user) [29].

A link to our webserver Missense3D (available at http://www.sbg.bio.ic.ac.uk/~missense3d/) that provides structural analysis of the effect of a missense variant is provided (manuscript under revision in JMB).

### Protein Information (summary) panel

The protein Information panel provides a high-level view of the data available for the protein of interest, such as the number of isoforms, variants, interactions and available experimental and model structures.

### Isoform panel

The isoform panel presents a list of available isoforms for a given protein. The data are retrieved from UniProt and presented “as-is.” Each alternative amino acid sequence is identified by its Uniprot Id, and the amino acid sequence displayed in the PhyreRisk page is highlighted. The Isoforms panel is dynamic and it allows selecting a protein isoform of interest and being redirected to the corresponding PhyreRisk page.

### Variants panel and Interactions panel

The *Variants* panel presents a list of variants currently stored in PhyreRisk database for the query protein derived from UniProt database. Therefore, this is not an exhaustive list of all known variants for a given protein.

The *Interactions* panel presents a list of known experimentally derived protein–protein interactions, supported by multiple observations from the IntAct database (as per UniProt).

### Documentation

PhyreRisk provides an extensive on-line tutorial available from the Home page and numerous tool tips.

## Appendix A Supplementary data

Supplementary material Image 1
