# Supplementary material for: PhyreRisk: A Dynamic Web Application to Bridge Genomics, Proteomics and 3D Structural Data to Guide Interpretation of Human Genetic Variants
Source: J Mol Biol. 2019 Jun 14;431(13):2460–6. doi: 10.1016/j.jmb.2019.04.043 (PMC6597944; doi:10.1016/j.jmb.2019.04.043)
Supplement: Supplementary file 1 — Supplementary material [file mmc1.doc]

**Supplemental material**

**PhyreRisk: a dynamic web application to bridge genomics, proteomics and 3D structural data to guide interpretation of human genetic variants**

*Tochukwu C. Ofoegbu (1), *Alessia David (1), Lawrence A. Kelley (1), Stefans Mezulis (1), Suhail A. Islam (1), Sophia F. Mersmann (1), Léonie Strömich (1), Ilya A. Vakser (2), Richard S. Houlston (3) and Michael J.E. Sternberg (1)

1. Centre for Integrative Systems Biology and Bioinformatics, Department of Life Sciences, Imperial College London, London, SW7 2AZ UK

2. Computational Biology Program and Department of Molecular Biosciences, The University of Kansas, Lawrence, Kansas 66045, USA

3. Division of Genetics and Epidemiology, The Institute of Cancer Research, London, SM2 5NG,

UK

- **Supplementary Figures**
- **The JSmol console**

1. ***How to visualize multiple residues using the JSmol console***
2. ***Examples of commands that can be used from the JSmol console for model structures***
3. ***Examples of commands that can be used from the JSmol console for PDB structures***

**Suppl Figure 1 PhyreRisk Genetic Variants output page with link into protein page**

**Suppl Figure 2 PhyreRisk Protein Variants Input page**

**Suppl Figure 3 Example of PhyreRisk search page**

***The JSmol console***

1. ***How to visualize multiple residues using the JSmol console***
2. ***Examples of commands that can be used from the JSmol console for model structures***
3. ***Examples of commands that can be used from the JSmol console for PDB structures***

***1. How to visualize multiple residues using the JSmol console***

**On the JSmol structure viewer, right click using the mouse. This will display a list of options:**


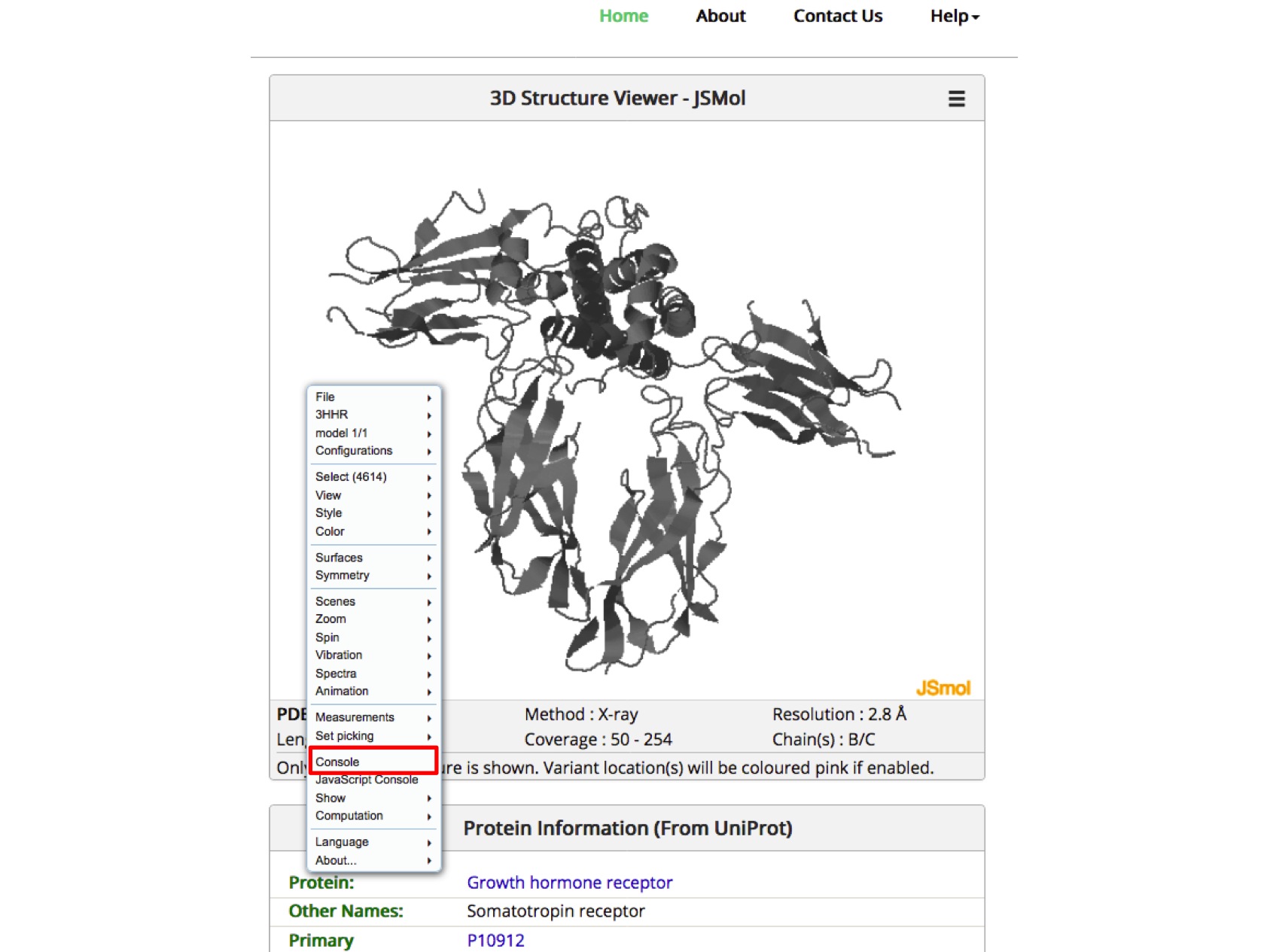


**Select console. The JSmol console will open and will look like this:**


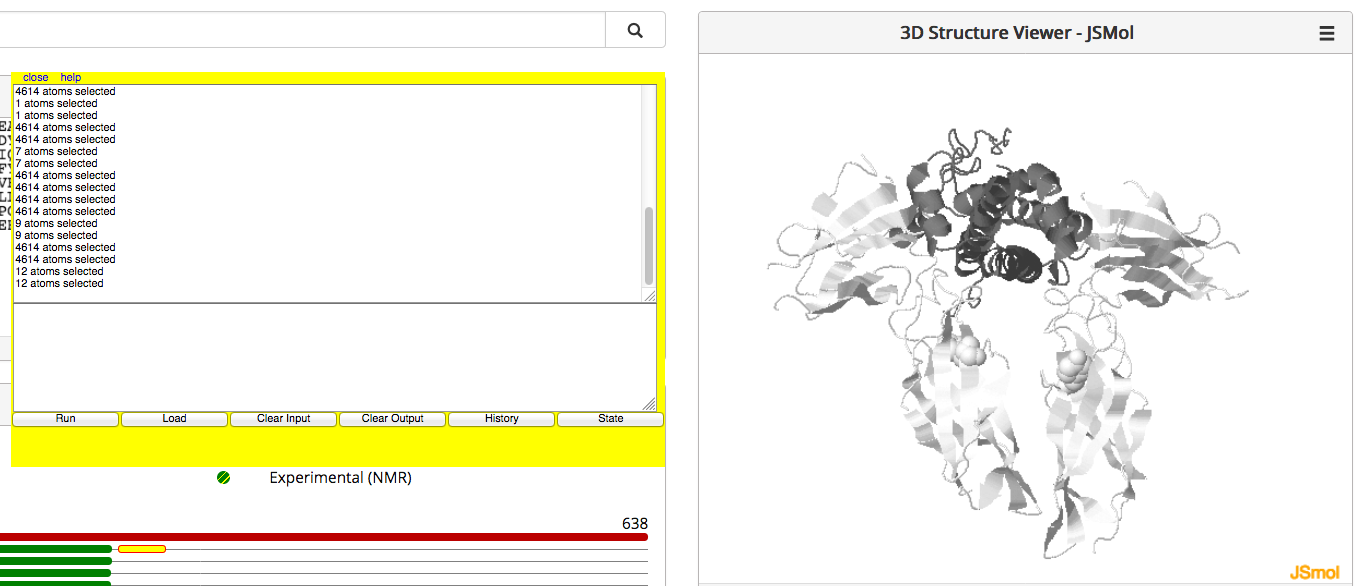


***2. Examples of commands that can be used from the JSmol console for model structures***

**Displaying residues in Phyre models is easier than using PDB structures. All Phyre models have been generated following the residue numbering in UniProt and no additional mapping is required. Moreover, all Phyre models are single chain. Therefore to select one or more residues of interest simply type:**

**select 60,70; color red; wireframe 1.5 (press Enter on your keyboard)**

**To change the background color to lightgrey, type the following command and then press Enter on your keyboard:**

**color background lightgrey (press Enter on your keyboard)**

**In the example below, the chain in the Phyre model corresponding to the growth hormone receptor, (modelled using the PDB ID 1HWH chain B as template) is shown before and after.**


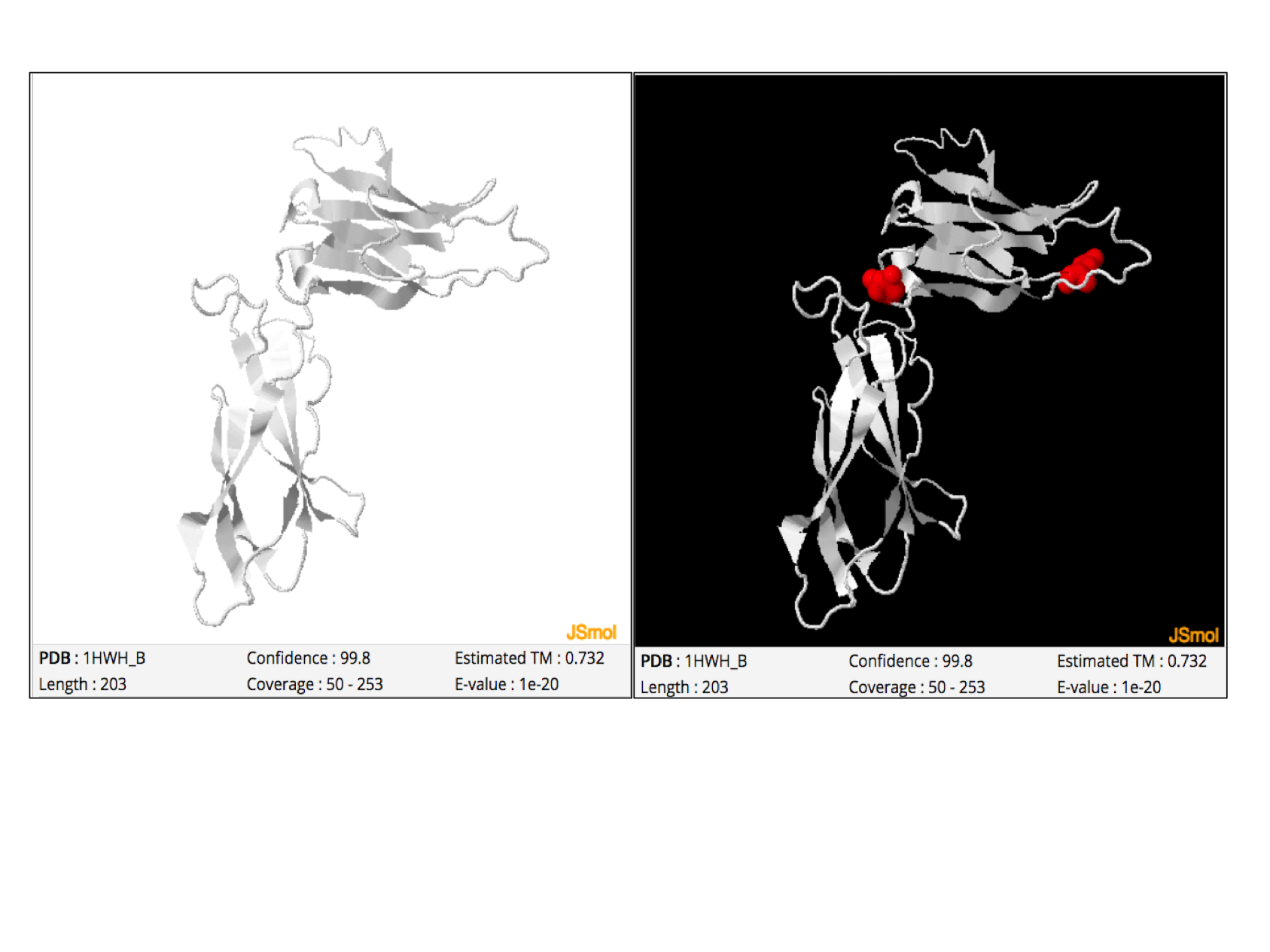


**To select everything, type the following command and then press Enter on your keyboard:**

**Select ***

**To show the sidechains of all residues, type the following command and then press Enter on your keyboard:**

**wireframe 0.5**

**(the number can be changed to modify the thickness in which the side chain is displayed; try wireframe 1.5 or wireframe 0.2 to look at different effects)**

**To hide all side chains, type the following command and then press Enter on your keyboard:**

**wireframe off**

***3. Examples of commands that can be used from the JSmol console for PDB structures***

***IMPORTANT: the numbering of amino acids in PDB structures is arbitrary and does not necessarily correspond to UniProt numbering. When using the console to display residues it is important to know the UniProt-to-PDB mapping as well as the chain that corresponds to the protein of interest.***

**To select chain A only, type the following command and then press Enter on your keyboard:**

**select :a**

**To color the chain you have selected in lightgreen, type the following command:**

**color lightgreen (press Enter on your keyboard)**

**In the example below, the three chains (a, b, c) in PDB ID: 3hhr corresponding to the growth hormone receptor dimer (chains b and c) bound to growth hormone (chain a) have been colored in pink, yellow and lightgreen and the background in black.**


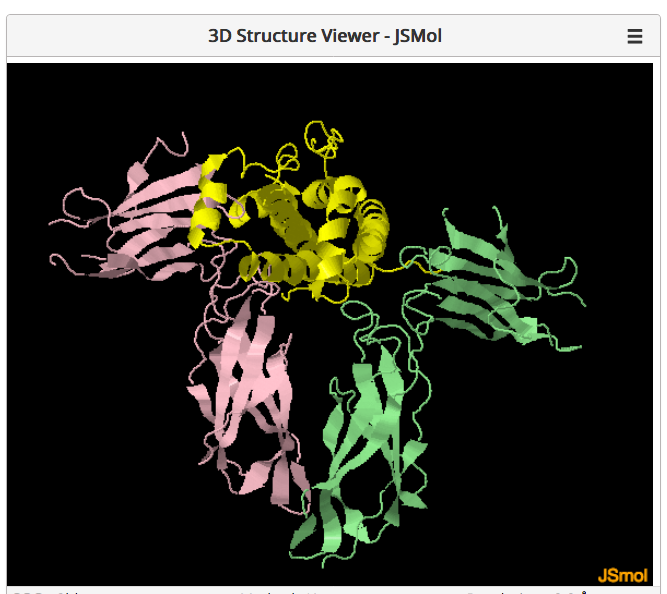


**To select residue 40 in chain c of the PDB file, type:**

**select :c and 40 (press Enter on your keyboard)**

**To color this residue in red and display its side chain, type:**

**color red; wireframe 0.5 (press Enter on your keyboard)**

**The structure should now look like this:**


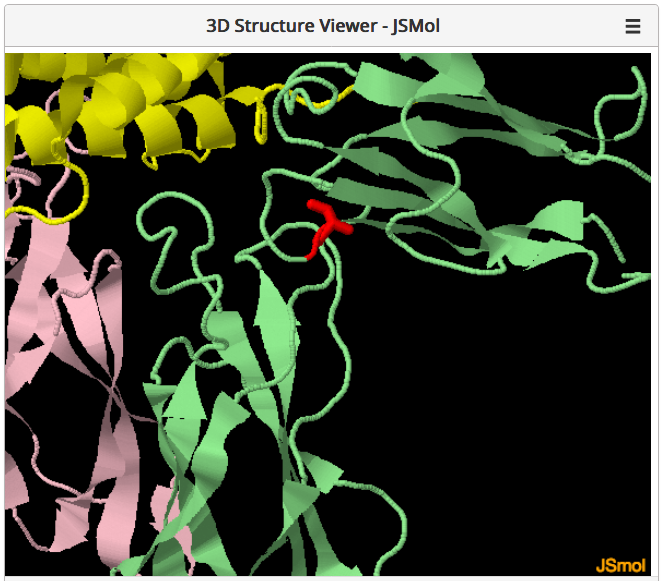


**To display multiple residues at once and color them in red, type:**

**select :c and 40,50; color red; wireframe 0.5 (press Enter on your keyboard)**

**The structure should now look like this:**


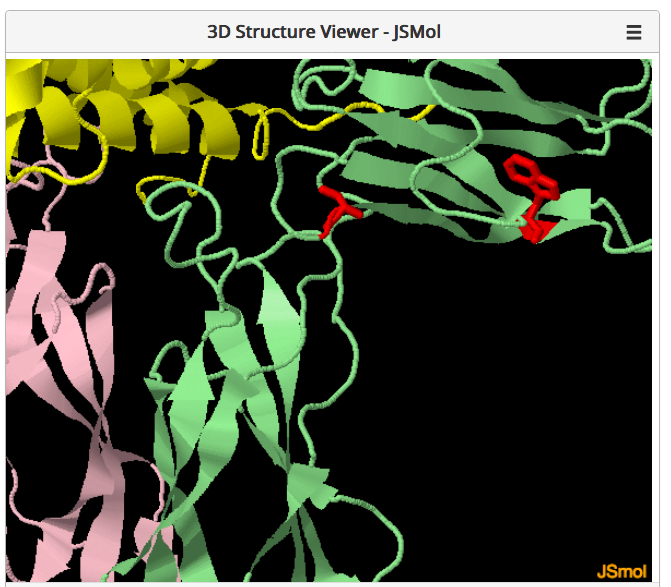


**To add the residue labels right click on the structure viewer, select “Color”, “Labels” and choose the color, in this case “White”. The picture should now look like this:**


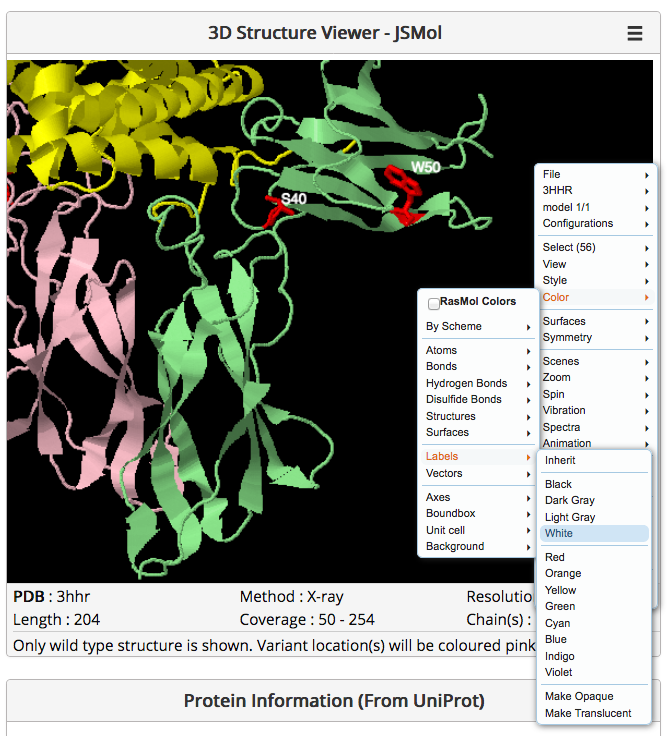


To display all proline residues on chain c, display their side chains and color them in red, type

select :c and pro **(press Enter on your keyboard)**

wireframe 0.5 **(press Enter on your keyboard)**

color red **(press Enter on your keyboard)**

The picture should now look like this:


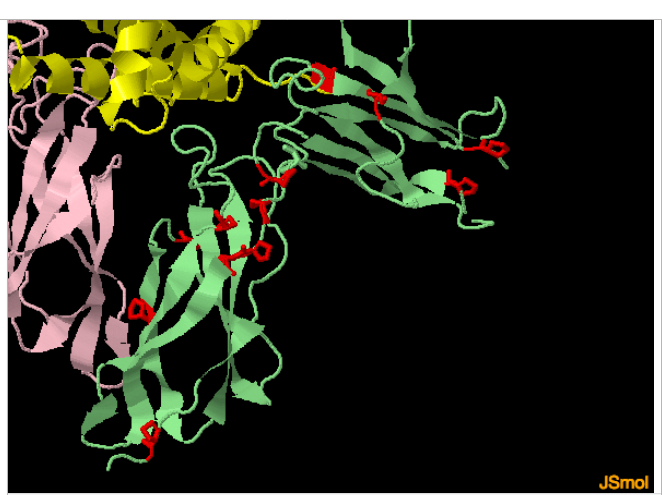


To select all hydrophobic residues, type and then **press Enter on your keyboard**:

select hydrophobic

**Additional and more advanced command lines can be found on the web. However, PhyreRisk is not designed to be used as a molecular viewer and for such a task we recommend using Pymol or EzMol molecular viewers.**
